# 190. Meta-analysis of Impact of Follow-up Blood Cultures on Mortality in Patients with Gram-negative Bacteremia

**DOI:** 10.1093/ofid/ofad500.263

**Published:** 2023-11-27

**Authors:** Khalid Eljaaly

**Affiliations:** King Abdulaziz University, Saudi Arabia, Jeddah, Saudi Arabia

## Abstract

**Background:**

It is not clear if follow-up blood cultures are beneficial in patients with Gram-negative bacteremia. It may improve clinical outcomes but have some drawbacks such as the possibility of inaccurate positive findings and extending the length of hospital stay and duration of antibiotic therapy. The aim of this meta-analysis was to evaluate the impact of follow-up blood cultures on mortality in patients with Gram-negative bacteremia.

**Methods:**

PubMed and EMBASE databases were searched from inception to Jan 2023 for randomized controlled trials or observational studies comparing the mortality in patients with Gram-negative bacteremia who had follow-up blood cultures obtained versus those without these cultures. We estimated odds ratios (ORs) with 95% confidence intervals (CIs) using random-effects models and assessed for heterogeneity (I^2^).

**Results:**

A total of six observational studies were included, while no randomized controlled trials were identified. The total number of patients was 4,631. One study used 28-day mortality, two studies used 30-day mortality, and three studies used in-hospital mortality. Follow-up blood cultures were associated with significantly lower odds of mortality ((OR, 0.6; 95%CI, 0.5-0.7; I^2^, 0%). No publication bias or heterogeneity was observed.

**Conclusion:**

The use of follow-up blood cultures in patients with Gram-negative bacteremia was associated with lower mortality. Randomized controlled trials are needed. Currently, follow-up blood cultures should be obtained for at least the high-risk patients with Gram-negative bacteremia.

**Disclosures:**

**Khalid Eljaaly, PharmD, MS, BCPS, BCIDP, FCCP**, Reckitt Benckiser Healthcare International Ltd, UK: Member of the Global Respiratory Infection Partnership, supported by unrestricted educational grant from Reckitt Benckiser Healthcare Ltd

